# Silicate/zinc-substituted strontium apatite coating improves the osteoinductive properties of β-tricalcium phosphate bone graft substitute

**DOI:** 10.1186/s12891-021-04563-4

**Published:** 2021-08-09

**Authors:** Hironori Sugimoto, Yusuke Inagaki, Akira Furukawa, Tsutomu Kira, Sachiko Kawasaki, Yoshinobu Uchihara, Manabu Akahane, Yasuhito Tanaka

**Affiliations:** 1 Department of Orthopaedic Surgery, Nara Medical University, Shijocho 840, 634-8521 Kashihara, Nara Japan; 2 Department of Health and Welfare Services, National Institute of Public Health, 2-3-6 Minami, 351-0197 Wako, Saitama Japan

**Keywords:** β-tricalcium phosphate, bone graft substitute, apatite coating, trace elements

## Abstract

**Background:**

β-Tricalcium phosphate (β-TCP) is a popular synthetic bone graft substitute with excellent osteoconductive properties and bioabsorbability. However, its osteoinductive properties are inferior to those of autologous or allogeneic bone. Trace elements such as strontium (Sr), silica (Si), and zinc (Zn) have been reported to promote osteogenesis in materials. In this study, we aimed to determine whether a Si/Zn-substituted Sr apatite coating of β-TCP could enhance osteoinductive properties.

**Methods:**

The apatite-coated β-TCP disks were prepared using nanoparticle suspensions of silicate-substituted Sr apatite (SrSiP) or silicate- and Zn-co-substituted Sr apatite (SrZnSiP).

Bone marrow mesenchymal cells (BMSCs) from rat femur were cultured and subsequently seeded at a density of 1.0 × 10^6^/cm^2^ onto apatite-coated and non-coated β-TCP disks.

*In vitro*, the β-TCP disks were then placed in osteogenic medium, and lactate dehydrogenase (LDH) activity was measured from supernatants after culture for 2 days. Additionally, after culture for 14 days, the mRNA expression of genes encoding osteocalcin (OC), alkaline phosphatase (ALP), bone morphogenetic protein-2 (BMP-2), and vascular endothelial growth factor (VEGF) was evaluated by qRT-PCR. *In vivo*, the β-TCP disks were transplanted subcutaneously into rats that were sacrificed after 4 weeks. Then, the harvested disks were evaluated biochemically (ALP activity, OC content, mRNA expression of OC, ALP, BMP-2, and VEGF measured by qRT-PCR), radiologically, and histologically.

**Results:**

Significantly higher mRNA expression of almost all evaluated osteogenic and angiogenic genes was observed in the SrZnSiP and SrSiP groups than in the non-coated group, with no significant cytotoxicity elicited by the apatite coating in vitro. Moreover, in vivo, the SrZnSiP and SrSiP groups showed significantly higher osteogenic and angiogenic gene expression and higher ALP activity and OC content than the non-coated group (*P* < 0.05). Radiological and histopathological findings revealed abundant bone formation in the apatite-coated group.

**Conclusions:**

Our findings indicate that apatite coating of β-TCP improves osteoinductive properties without inducing significant cytotoxicity.

## Introduction

Calcium phosphate ceramics have excellent biocompatibility because their composition is similar to that of natural bone, making them ideal and widely used synthetic bone graft substitutes in the field of orthopedic surgery [1, 2]. Recently the synthetic bone grafts are utilized to stabilize the intended bone defects from surgeries such as the high tibial osteotomy for knee osteoarthritis [3, 4]. Among the commonly employed calcium phosphate ceramics, β-tricalcium phosphate (β-TCP) and hydroxyapatite (HA) are representative synthetic bones. In particular, β-TCP has high porosity and improved osteoconductive properties with a solubility that is approximately 30-fold higher than that of HA, making it more bioabsorbable and convenient for clinical application [5]. In addition, β-TCP grafts have a functional life expectancy of 12 to 16 months, and generally, they are completely replaced by natural bone within 3 years of grafting [6]. However, β-TCP has minimal or no osteoinductive properties, which stimulate bone marrow mesenchymal cells (BMSCs) to differentiate into osteoblasts and chondroblasts; thus, its osteogenic capacity is limited compared with that of autologous or allogenic bone grafts [1, 7].

To overcome this disadvantage, we have reported numerous methods involving loading cultured cells and cell sheets of BMSCs onto β-TCP [7–10], and such a method has already been applied for the treatment of various conditions, including avascular necrosis of the femoral head [11]. However, the preparation of these cell products is laborious, complex, and inconvenient.

Previous studies have also reported the use of growth factors as an alternative method. Indeed, osteogenesis can be enhanced by loading several growth factors, including bone morphogenetic protein-2 (BMP-2) and vascular endothelial growth factor (VEGF), onto β-TCP [12, 13]. Although most results have been favorable, these growth factors have been under the surveillance by the Food and Drug Administration owing to potentially serious side effects following clinical application [14, 15]. Therefore, β-TCP possessing its own osteoinductive properties with improved convenience and safety is clinically desirable.

As a suitable approach to enhance the osteoinductive properties of β-TCP, the inclusion of trace elements, such as Sr, Si, Mg, and Zn, which are essential for bone formation, has been investigated [16–19]. On the basis of these progress, we have developed a technology for nano-coating these trace elements onto various materials [20]. For example, the nano-coating polyethylene terephthalate (PET) artificial ligament and polyether-ether-ketone (PEEK) disks with silicate-substituted strontium apatite (SrSiP), or silicate- and zinc-co-substituted strontium apatite (SrZnSiP) enhances osteoinductive properties and promotes osteogenesis [21, 22].

Therefore, the purpose of the current study was to determine whether the nano-coating β-TCP with SrSiP or SrZnSiP improves its osteoinductive properties and promotes osteogenesis both in vitro and in vivo.

## Methods

### Strontium apatite-coated β-TCP disks

The substituted strontium apatite used herein has been described in detail previously [23]. Briefly, silicate-substituted SrSiP was synthesized from Sr hydrogen phosphate and sodium metasilicate, and SrZnSiP was prepared by the partial ion-exchange reaction of SrSiP with zinc chloride. A strontium apatite coating solution was prepared by wet milling; strontium apatite powder was shaken with 0.3 mm φ zirconium balls (14 % solid) in ethanol overnight to yield nano-sized particle dispersion, which was diluted with 1 % ethanol. Sterilized Superpore porous β-TCP disks with interconnecting pores (diameter, 5 mm; thickness, 2 mm; porosity, 60 %; macro pore diameter, 50 ~ 300 μm; micro pore diameter, under 10 μm.) (Hoya Corp., Tokyo, Japan) and each apatite solution (1 wt%) was placed in 10 cc injection syringes (Terumo, Tokyo, Japan).

Air bubbles inside the β-TCP disks were aspirated under negative pressure to ensure the apatite solution was applied to the entire β-TCP disks. Excess solution was removed, and the coated β-TCP disks were heated at 300 °C for 72 h. The apatite solution concentration used (1 wt%) was　based on preliminary experiments with various concentrations.

We defined three groups: SrZnSiP, SrSiP and control (comprising non-coated normal β-TCP disks). Samples from the three groups were examined using an SU3500 low-vacuum scanning electron microscope (SEM; Hitachi Ltd., Tokyo, Japan), equipped with an Octane Plus energy dispersive X-ray spectrometer (EDS; Ametek Inc., Mahwah, NJ, U.S.A.) at an acceleration voltage of 20 kV at 60 Pa. The elemental composition of the apatite coating on the disks was analyzed using EDS and their microarchitecture was observed at 30⋅ and 200⋅ magnification using SEM.

### Sr apatite-coated β-TCP disk-bone marrow-derived mesenchymal cell constructs

 All experimental procedures using animals were approved by the institutional animal care and use committee (approval number 12,731). The study complied with the standards of the National Institutes of Health and the ARRIVE guidelines.

A total of 20 rats were housed at ~ 21 °C under a 12-hour light/dark cycle, with free access to food and water. BMSCs were prepared as previously described [9, 24].

Briefly, 7-week-old male Fisher-344 rats (SLC Japan Inc., Shizuoka, Japan) were euthanized by 4 % isoflurane (Pfizer, Puurs, Belgium) inhalation for 5 min inside a sealed container. In addition, 50 mg/mL pentobarbital sodium salt (Tokyo Chemical, Tokyo, Japan) was injected into the peritoneal cavity. Death was confirmed based on cardiac arrest, respiratory arrest, and loss of corneal reflexes. Bone marrow cells from bilateral femoral bones were collected in 75 cm^2^ culture flasks (Falcon; BD Biosciences, San Jose, CA, USA) containing 15 mL of regular medium, comprising minimal essential medium (Nacalai Tesque, Kyoto, Japan) supplemented with 15 % fetal bovine serum (Sigma-Aldrich, St. Louis, MO, USA) and antibiotics (100 U/mL penicillin and 100 µg/mL streptomycin; Nacalai Tesque). Cells were cultured in a humidified atmosphere of 95 % air and 5 % CO_2_ at 37 °C. Non-adherent cells were removed three times weekly during medium changes.

Confluent primary cultured BMSCs were harvested after 14 days using trypsin (2.5 g/L)-ethylenediaminetetraacetic acid (EDTA; 1 mmol/L; Nacalai Tesque, Kyoto, Japan). The cells were then suspended in regular medium at a density of 1 × 10^6^/cm^2^. The β-TCP disks for each group were immersed in a suspension of BMSCs (1 × 10^6^ cells/cm^2^) in syringes (Terumo, Tokyo, Japan), for 2 h under CO_2_ atmosphere at 37 °C.

For the single series of *in vitro* and *in vivo* experiments described below, we prepared 19 disks for each group. Additionally, three rats were needed to obtain sufficient cell suspension for the single series experiments, which were performed twice. Hence, six rats were sacrificed to prepare cell suspensions.

### Experiment 1. Comparison of SrZnSiP, SrSiP, and control groups in vitro

#### Osteogenic culture of each β-TCP disk-bone marrow-derived mesenchymal cell construct

We placed one β-TCP disk with BMSCs in each well of 12-well Falcon plates (BD Biosciences) in regular osteogenic medium containing 10 nmol/L dexamethasone (Sigma, St. Louis, MO, USA), 0.28 mmol/L l-ascorbic acid magnesium phosphate salt n-hydrate (Wako Pure Chemical Industrials, Kyoto, Japan), and 10 mmol/L β-glycerol phosphate disodium salt pentahydrate (Sigma-Aldrich Corp., St. Louis, MO, USA). The medium was changed three times per week.

#### Cell cytotoxicity assays

Cytotoxicity resulting from apatite coating was evaluated as lactate dehydrogenase (LDH) activity, which was measured using an LDH Cytotoxicity Detection Kit (Takara Bio Inc., Kusatsu, Japan). Supernatants (n = 5) were collected after culture for 2 days, before the first medium change and transferred to new tubes. LDH activity was measured by determining absorbance at 490 nm with an automated microplate reader (SpectraMax M2, Molecular Devices, San Jose, CA, USA). LDH Standard Solution (Roche Holdings AG, Basel, Switzerland) was used for the standard.

#### Quantitative real-time polymerase chain reaction (qRT-PCR)

We disrupted β-TCP constructs after osteogenic culture for 14 days with 1 mL of TRIzol (Invitrogen; Thermo Scientific Inc., Waltham, MA, USA), using a micro-homogenizer at room temperature. The homogenate was transferred to a QIA shredder spin column (Qiagen Inc., Valencia, CA, USA), centrifuged at 17,700 ⋅ *g* for 2 min at room temperature, filtered, and thoroughly mixed with 200 µL of chloroform in new 1.5-mL microtubes. After 5 min at room temperature, the mixture was centrifuged at 17,700 ⋅ *g* for 15 min at 4 °C; the upper aqueous layer was then thoroughly mixed with 70 % ethanol in new 1.5-mL microtubes. RNA was harvested from samples using RNeasy Micro Kits (Qiagen, Hilden, Germany). It was reverse transcribed to cDNA using High-Capacity cDNA Reverse Transcription Kit with RNase Inhibitor (Thermo Fisher Scientific, Waltham, MA, USA) according to the manufacturer’s instructions, and a StepOnePlus Real-Time PCR System (Applied Biosystems, Foster City, CA, USA) was used to perform the PCR. The thermal cycling conditions were 20 s at 95 °C for activation of the TaqMan Fast Universal PCR Master Mix, followed by 40 cycles of 1 s at 95 °C for denaturing and 20 s at 60 °C for annealing and extension.

The mRNA expression of genes encoding osteocalcin (OC), alkaline phosphatase (ALP), bone morphogenetic protein-2 (BMP-2), and vascular endothelial growth factor (VEGF) was evaluated using a set of TaqMan® primers and probes, as well as OC (Rn01455285_g1), ALP (Rn00564931_m1), BMP-2 (Rn00567818_m1), and VEGF (Rn01511602_m1) assays kits (all from Thermo Fisher). Target mRNA levels were compared after normalization (*n* = 5) to those of the internal standard, glyceraldehyde-3-phosphate dehydrogenase (GAPDH, Rn99999916_s1; Thermo Fisher) [22, 25].

### Experiment 2. Comparison between SrZnSiP, SrSiP, and control groups in vivo

#### Ectopic transplantation of β-TCP disks into the subcutaneous pockets on the backs of rats

**Fig. 1:**
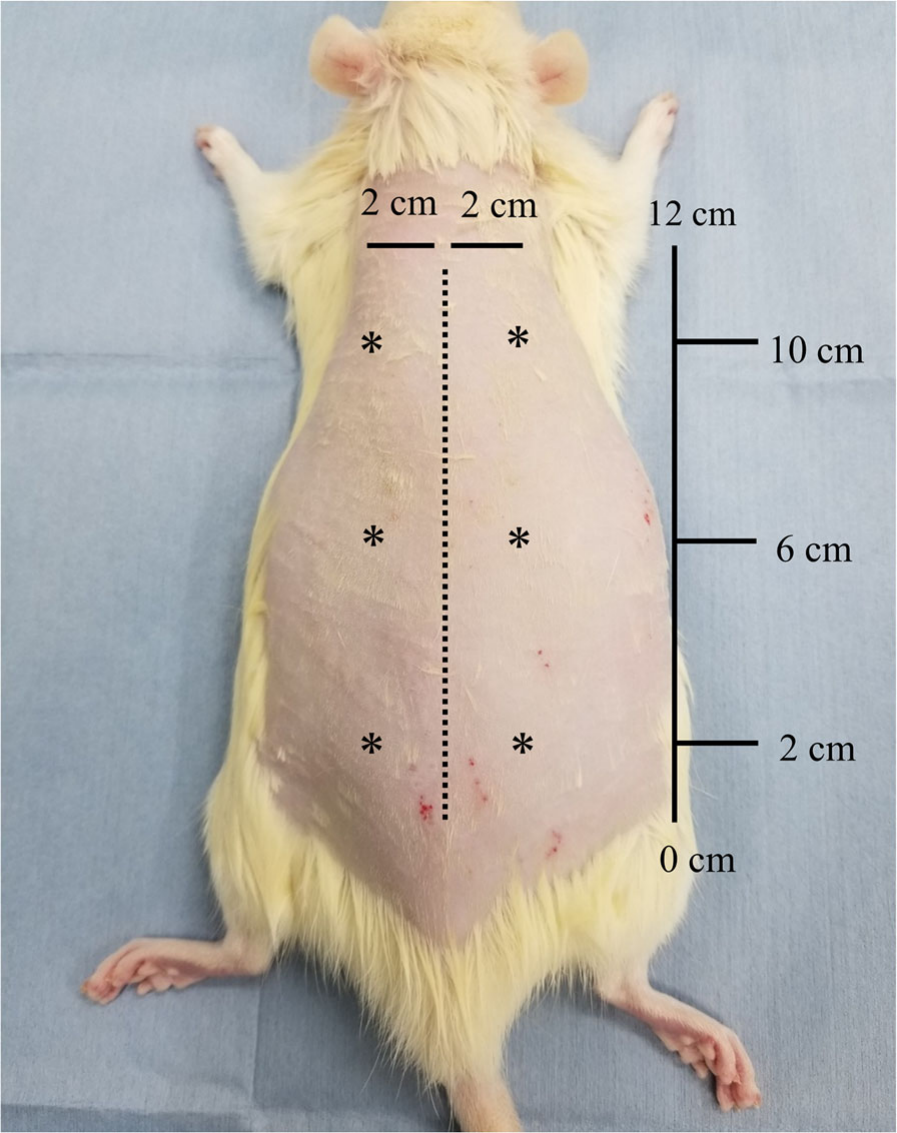
Transplantation points of β-TCP disks. Each point is indicated by an asterisk

Each β-TCP disk with BMSCs was implanted into a subcutaneous pocket on the back of a syngeneic 7-week-old male rat, as previously described [26, 27]. For uniform subcutaneous transplantation, we set three points (2 cm, 6 cm, and 10 cm) along the 12 cm long axis, with symmetrical positions from the center line, to enable a total of six β-TCP disks, two disks per group, to be implanted in each rat (Fig. 1). Seven rats were used for subcutaneous implantation in each experiment. The *in vivo* procedures described below were conducted twice (total number of rats, *n* = 14). The transplanted β-TCP disks were harvested after 4 weeks and evaluated biochemically, radiologically, and histologically.

#### Biochemical evaluation

##### Quantitative RT-PCR

We extracted RNA from harvested β-TCP disks, as described above. The mRNA expression of genes encoding OC, ALP, BMP-2, and VEGF was evaluated, as mentioned above.

##### ALP activity and OC content

We measured ALP activity as a key differentiation marker of the osteoblast phenotype and OC content to assess the osteogenic potential of BMSCs, as described previously [8] [28] [29]. The β-TCP constructs were homogenized in 1 mL of 0.2 % Nonidet P-40 (NP-40) and centrifuged at 11,000 ⋅ *g* for 10 min at 4 °C. Supernatants (10 µL) were incubated with 56 mmol/L amino-2-methylpropane diol buffer containing 10 mmol/L p-nitrophenyl phosphate and 1 mmol/L MgCl_2_ for 30 min at 37 °C; the reaction was stopped by adding 0.2 N NaOH. The ALP activity was calculated from the absorbance of p-nitrophenol released at 410 nm, as determined by spectrophotometry (*n* = 5). OC content was isolated from sediment after 2 weeks of extraction with 0.2 % NP-40 in 4 mL of 20 % formic acid at 4 °C. Extracts (0.5 mL) were eluted through NAP-5 packed Sephadex G-25 columns (Amersham Pharmacia Biotech AB, Uppsala, Sweden) with 10 % formic acid. Protein fractions were then pooled and dried. OC content was assayed in fractions (*n* = 5) and dissolved in the sample buffer (0.5 mL) provided with the Rat Osteocalcin ELISA kit (DS Pharma Biomedical Co., Ltd., Osaka, Japan).

#### Radiological and histological evaluation

##### Micro-CT analysis

Harvested β-TCP disks were fixed in 10 % neutral buffered formalin (Wako Pure Chemical), and subsequently scanned using a CosmoScan FX high-resolution Micro-CT instrument (Rigaku Corp., Tokyo, Japan) at 360° rotation. The X-ray source was set at 90 kVp energy and 88 mA intensity, with an isotropic voxel size of 10 μm, and an aluminum/copper filter (Al/Cu, 0.5/0.06), producing an image matrix of 512 × 512 pixels. We assessed the mineralized bone tissue area in each group using a centered Micro-CT image of 2 mm thick β-TCP disks selected as the region of interest. Based on previous studies [30] [31], the mineralized bone tissue area was defined by a threshold of 550 and an upper threshold of 2000 in all samples, as calculated using the ImageJ color-threshold analyzer (National Institutes of Health, Bethesda, MD) (*n* = 4).

##### Histological analysis

For histological evaluation, after radiological evaluation, two samples were randomly selected from each group and decalcified in 10 % EDTA/phosphate-buffered saline (Wako Pure Chemical) for 2 days. Each sample was embedded in paraffin, the disk was cut through the middle, and histologically stained with hematoxylin and eosin (n = 2).

### Statistical analysis

Levels of mRNA expression, LDH and ALP activity, OC content, and areas of new bone formation were calculated and are presented as means ± standard deviation (SD). All data were statistically analyzed using one-way ANOVA with Tukey-Kramer post hoc tests for multiple comparisons by using SPSS ver. 22.0 (IBM Corp., Armonk. NY, USA). Values with *P* < 0.05 were considered statistically significant.

## Results

### SEM/EDS analysis of Sr apatite-coated β-TCP

**Fig. 2:**
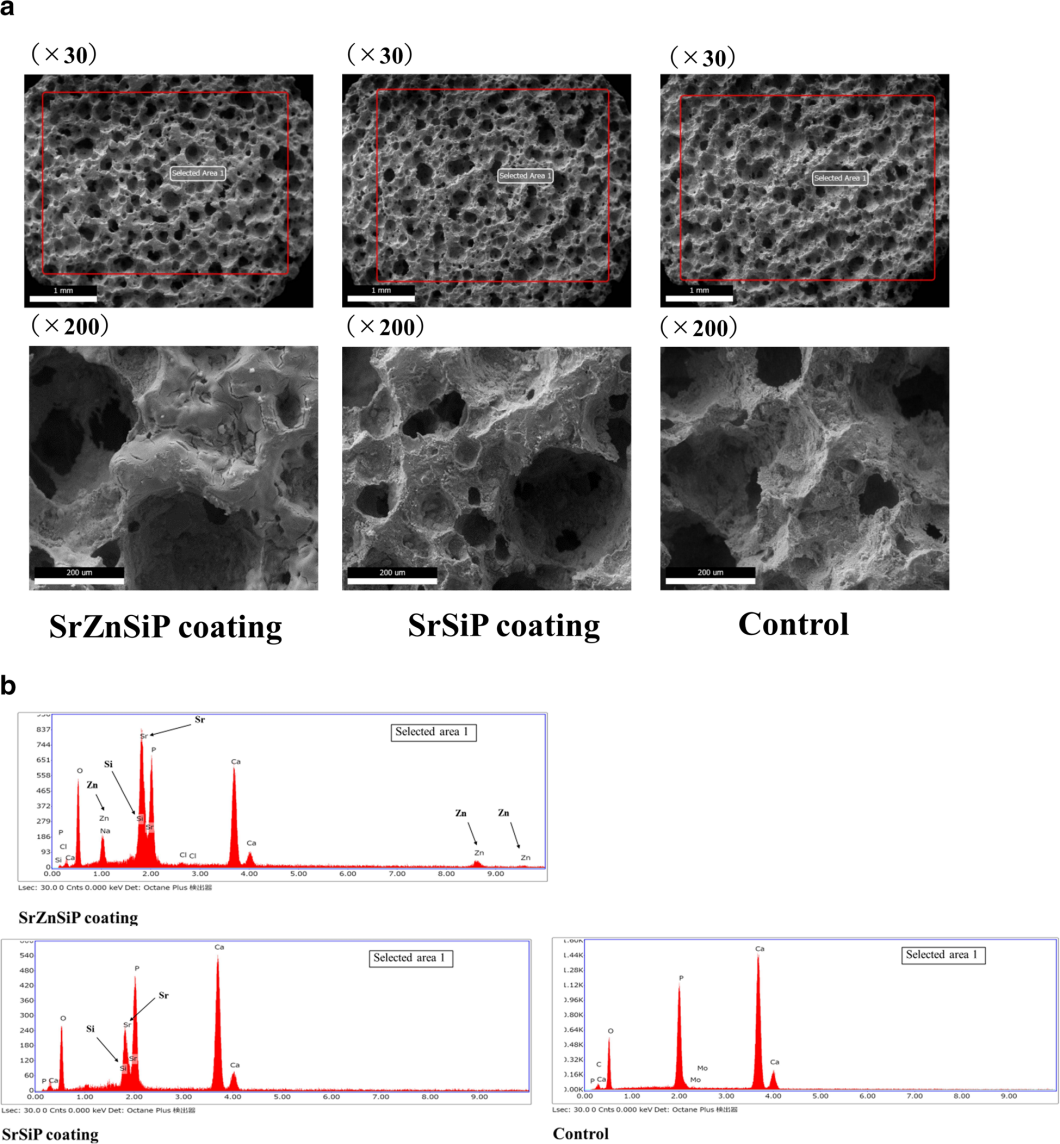
Representative samples of SrZnSiP, SrSiP, and control. Apatite coating analyzed by (**a**) SEM and (**b**) EDS. SEM, scanning electron microscopy; EDS, energy dispersive X-ray spectrometer

SEM of the SrZnSiP and SrSiP groups revealed that the pores of the β-TCP disks had not collapsed, and that the microarchitecture of the disks was retained after heating at high temperatures, similar to that of the control (Fig. 2a). EDS of the SrZnSiP and SrSiP groups showed appropriate distribution of Sr, Si, and Zn, confirming the validity of the apatite coating methodology (Fig. 2b).

### Experiment 1. In vitro study

#### Cell cytotoxicity assays in vitro

**Fig. 3:**
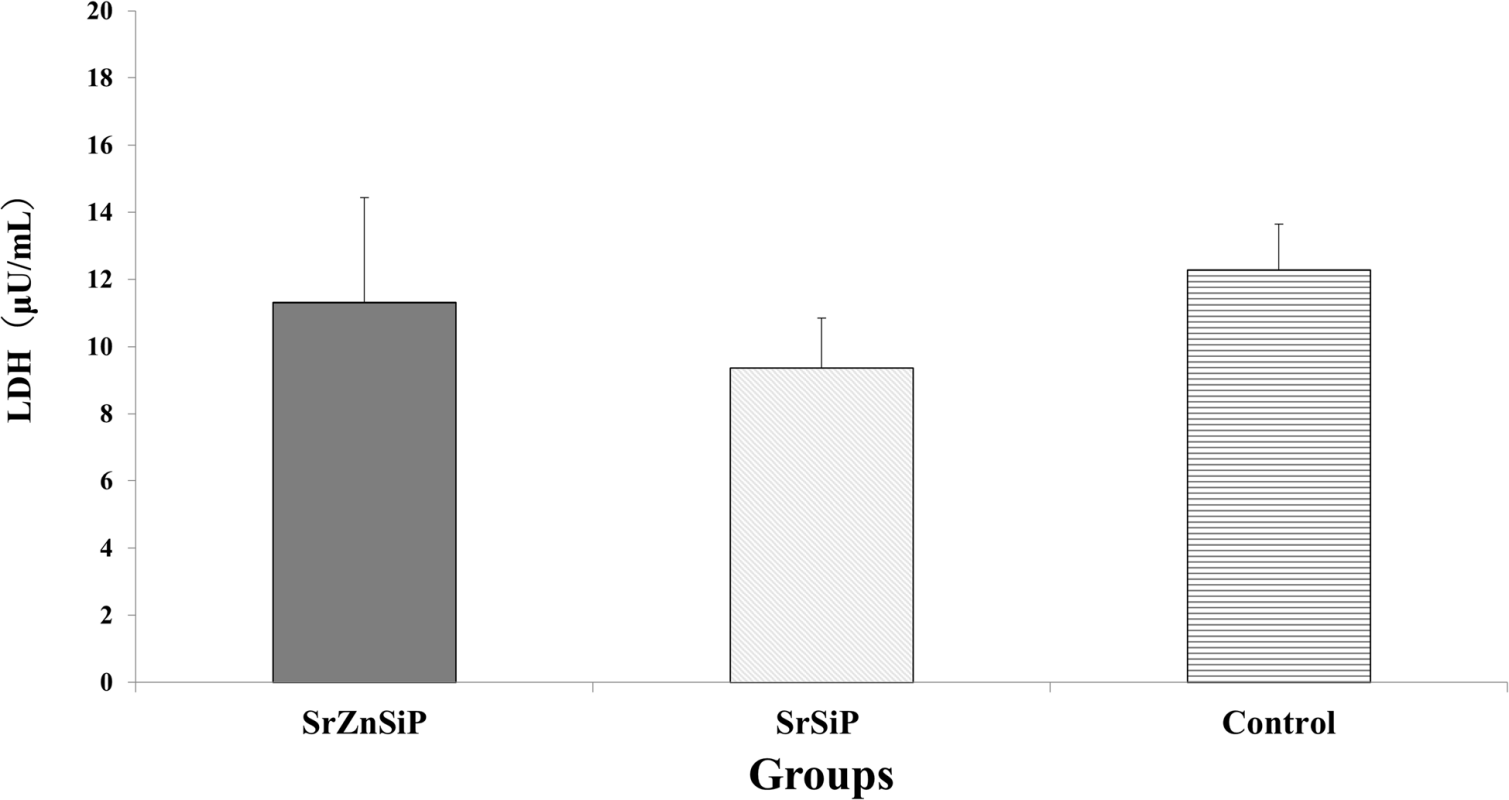
Activity of LDH secreted in media. Cell cytotoxicity assays using LDH showed no statistically significant differences among groups (*n* = 5). LDH, lactate dehydrogenase

There was no statistically significant difference in LDH values (SrZnSiP, SrSiP, and control groups; 11.31 ± 3.12, 9.36 ± 1.49, and 12.28 ± 1.36 µU/mL, respectively) among the three groups (*P* = 0.13). This result indicates that there is no cytotoxicity from apatite coating (Fig. 3).

#### Quantitative RT-PCR

**Fig. 4:**
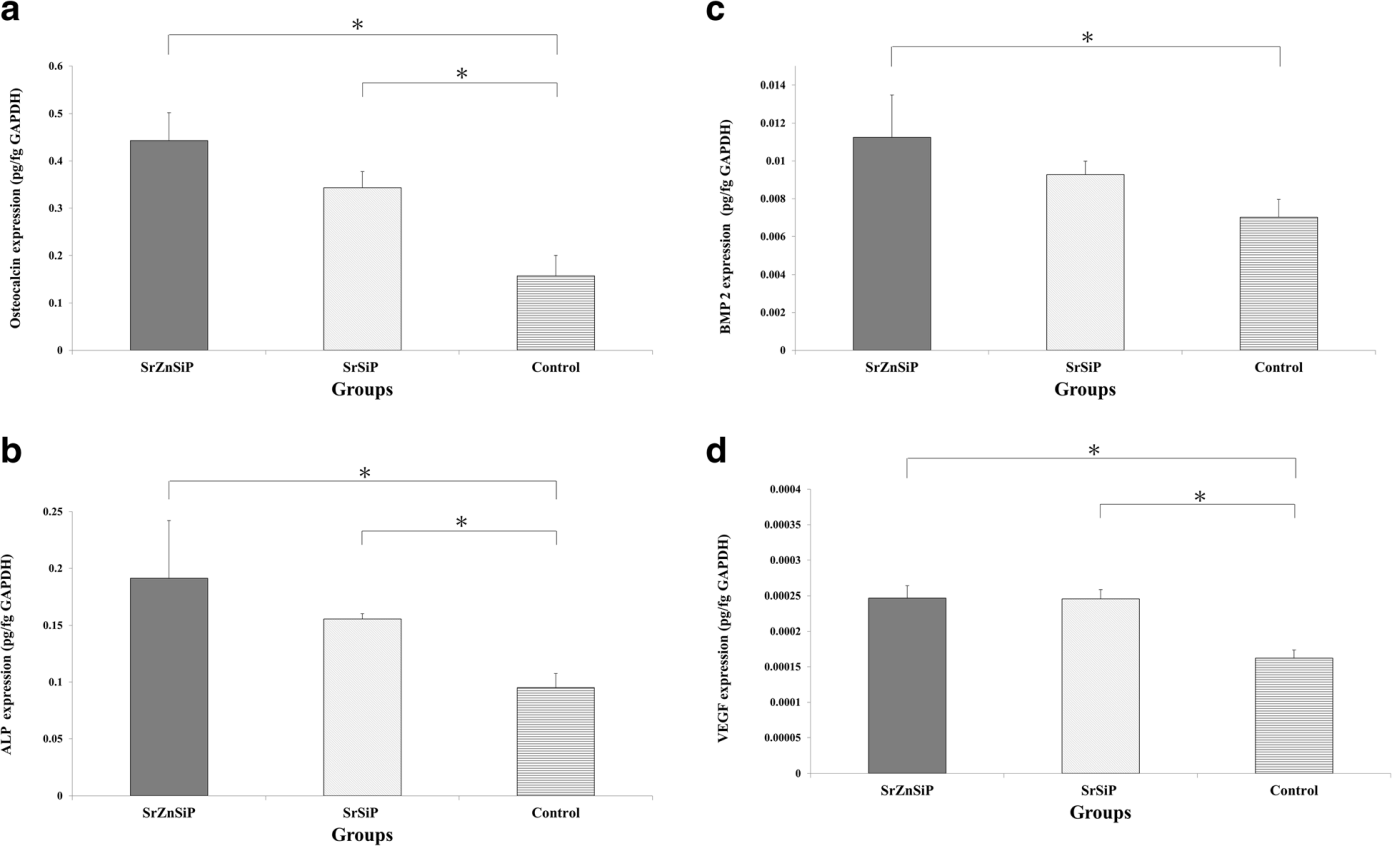
mRNA expression levels in each group after osteogenic culture for 14 days, as evaluated by qRT-PCR. Values are shown as means ± SD (n = 5). *p < 0.05. ALP, alkaline phosphatase; BMP-2, bone morphogenetic protein-2; OC, osteocalcin; qRT-PCR quantitative real-time polymerase chain reaction; VEGF, vascular endothelial growth factor

The mRNA levels of genes encoding OC, ALP, and VEGF were significantly higher in the apatite-coated group than in the control group (*P* < 0.05), and the mRNA levels of BMP-2 were significantly higher in the SrZnSiP group than in the control group (*P* < 0.05) (Fig. 4a and d).

There were no significant differences between the SrZnSiP and SrSiP groups in all parameters.

### Experimental part 2: In vivo study

#### Quantitative RT-PCR

**Fig. 5:**
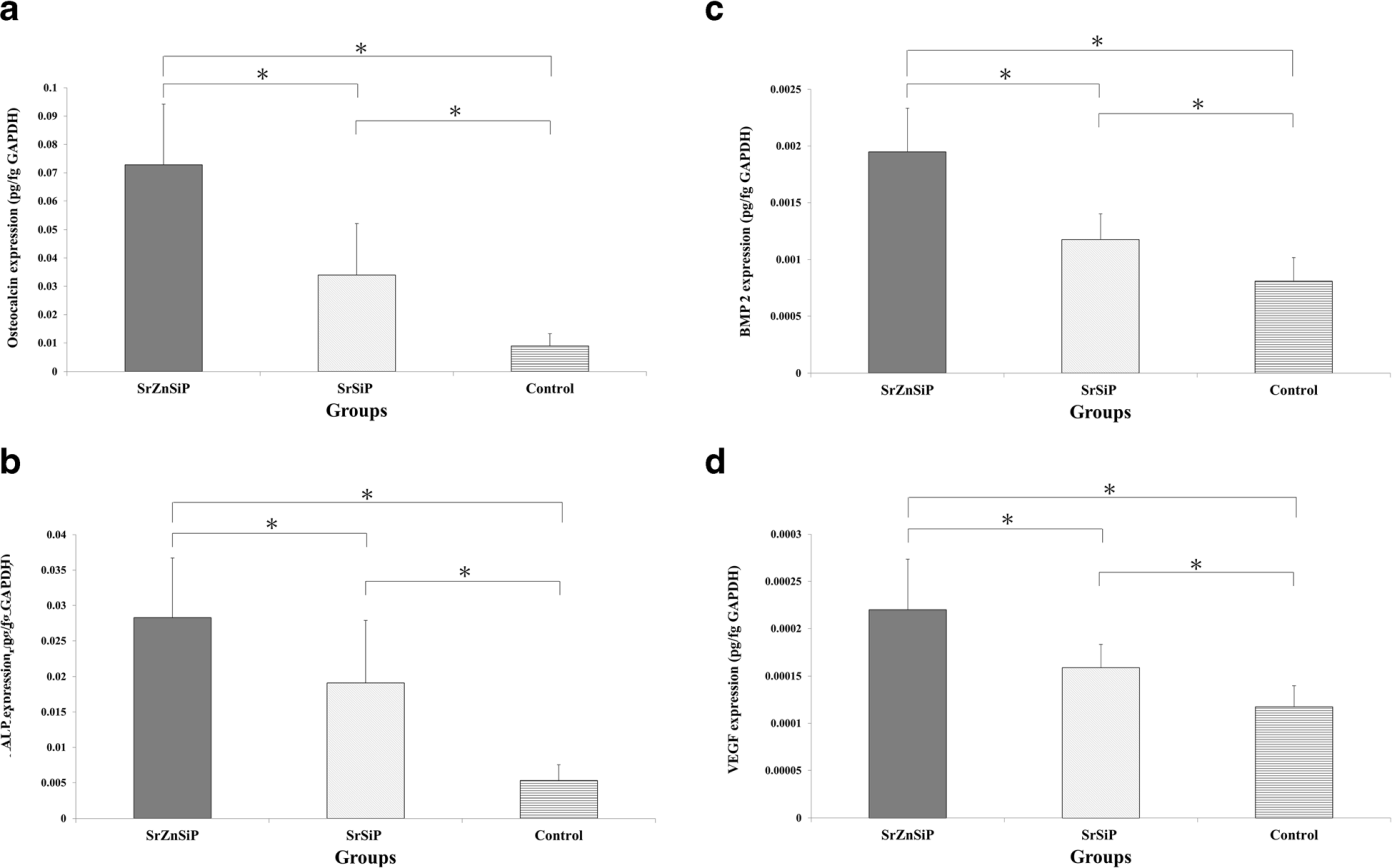
Expression of mRNA in groups of β-TCP harvested 4 weeks after implantation. Values are shown as means ± SD (*n* = 5). *p < 0.05. ALP, alkaline phosphatase; β-TCP, Beta-tricalcium phosphate; BMP-2, bone morphogenetic protein-2; OC, osteocalcin; qRT-PCR, quantitative real-time polymerase chain reaction; VEGF, vascular endothelial growth factor

The mRNA levels of genes encoding OC, ALP, BMP-2, and VEGF were significantly higher in the apatite-coated group than in the control group (*P* < 0.05) (Fig. 5a-d). In contrast to the trend observed *in vitro*, the mRNA levels of all parameters were significantly higher in the SrZnSiP group than in the SrSiP group (*P* < 0.05).

#### ALP activity and OC content

**Fig. 6:**
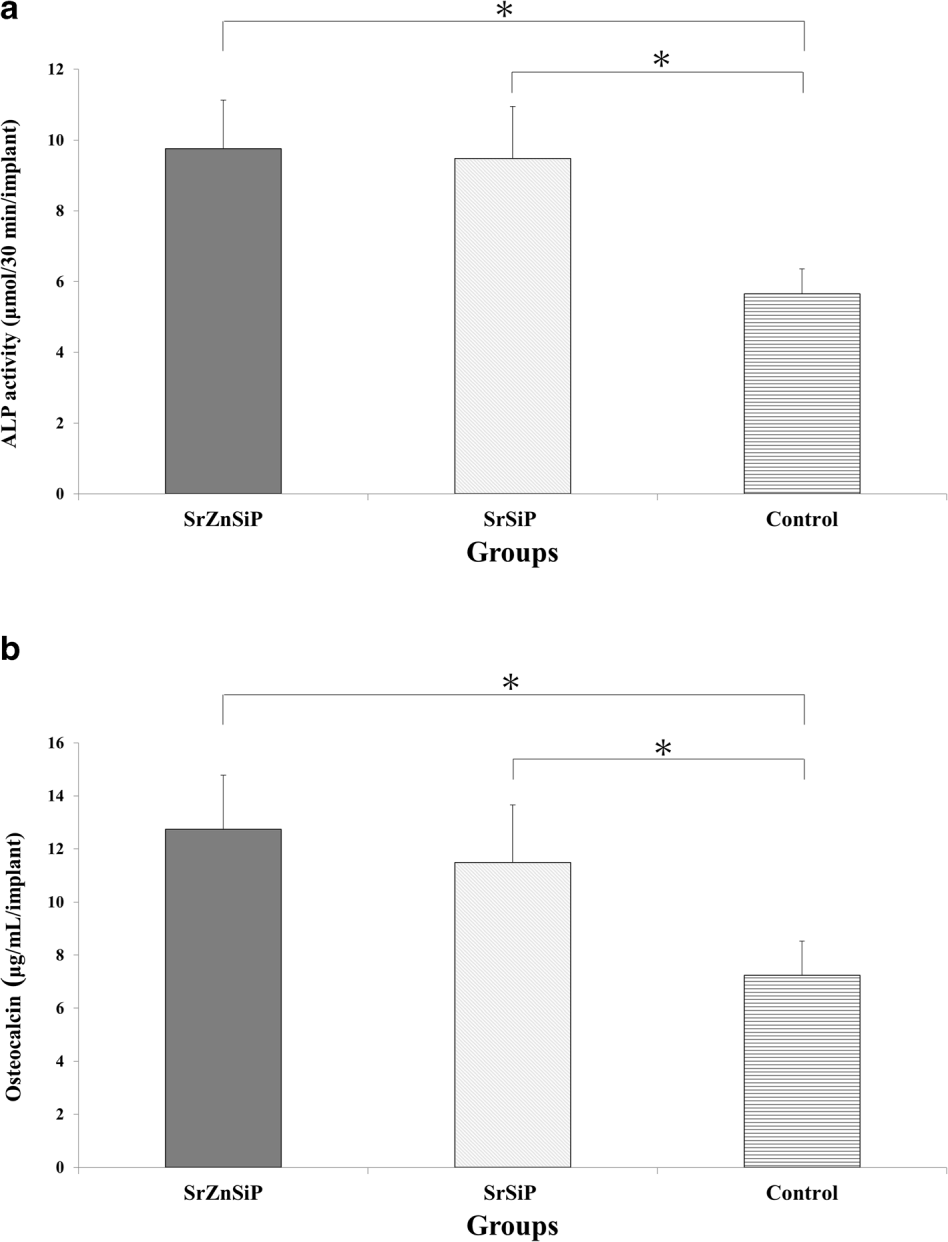
Biochemical evaluation of β-TCP harvested 4 weeks after implantation. ALP activity (**a**) and OC content (**b**). Both values are significantly higher in SrZnSiP and SrSiP than in controls (n = 5). Values are shown as means ± standard deviation. *p < 0.05. ALP, alkaline phosphatase; β-TCP, β-tricalcium phosphate; OC, osteocalcin

The ALP activity in the SrZnSiP, SrSiP, and control groups were, respectively, 9.75 ± 1.37, 9.47 ± 1.47, and 5.65 ± 0.70 µmol/30 min/implant and the OC content were, respectively, 12.73 ± 2.05, 11.49 ± 2.16 and 7.24 ± 1.28 µg/mL/implant. Both values were significantly higher in the apatite-coated group than in the control group (*P* < 0.05) (Fig. 6a and b).

#### Micro-CT analysis

**Fig. 7:**
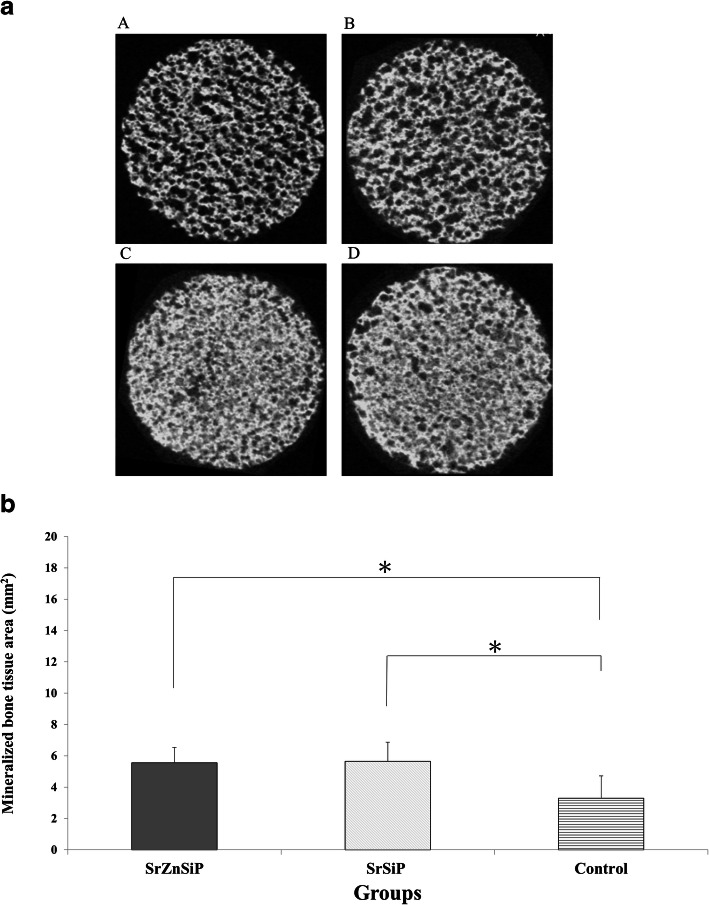
Micro-CT analysis of β-TCP harvested 4 weeks after implantation. (**a**) Representative images of all samples. A: pre-implantation, B: Control, C: SrSiP, D: SrZnSiP. (**b**) Mineralized bone tissue area for each group (*n* = 4). Micro-CT images demonstrated increased bone formation in the apatite-coated group. Values are shown as means ± SD (*n* = 5). *p < 0.05

At 4 weeks after transplantation, mineralized bone tissue was observed in the β-TCP void in all groups (Fig. 7a). The area of mineralized bone tissue bone was significantly higher in the SrZnSiP and SrSiP groups (5.55 ± 0.97, 5.64 ± 1.21 mm^2^, respectively) than in the control group (3.29 ± 1.42 mm^2^) (*P* < 0.05) (Fig. 7b).

#### Histological analysis

**Fig. 8:**
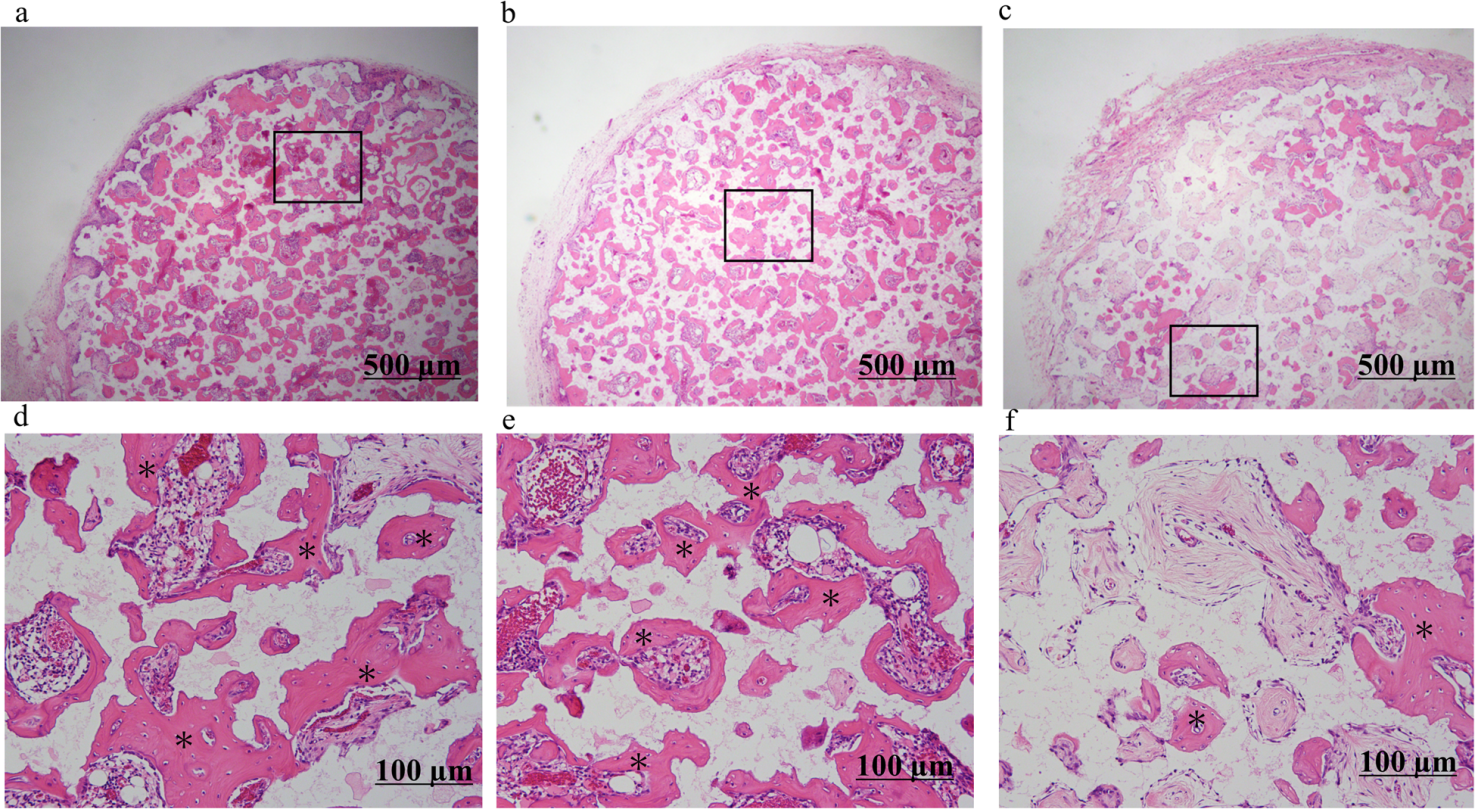
Histological analysis of β-TCP harvested 4 weeks after implantation. HE staining shows more bone formation in SrZnSiP and SrSiP than in controls. (**a**), SrZnSiP; (**b**), SrSiP; (**c**), control. (**d**–**f**) Higher magnifications of boxes indicated in (**a**), (**b**), and (**c**), respectively. * Bone tissue. β-TCP, β-tricalcium phosphate; HE, hematoxylin and eosin

β-TCP disks harvested from rats 4 weeks after transplantation were stained with hematoxylin and eosin. Higher levels of bone formation were observed in the apatite-coated groups than in the control group, indicating that apatite coating promoted osteogenesis (Fig. 8a-f).

## Discussion

One of the primary limitations associated with the clinical application of β-TCP as a bone implant substitute is its low osteoinductive capacity, which results in insufficient osteogenesis. Among the various methods to enhance the osteoinductive properties of β-TCP [7, 8, 32], the addition of bioactive trace elements, which play critical roles in bone formation, growth, and repair, is the safest, with fewer associated adverse effects. Indeed, the addition of these trace elements to bone scaffold materials has been shown to promote the formation of new bone *in vivo* [19, 33], control material degradation, and enhance mechanical strength [34, 35].

One such trace element is Sr, which stimulates osteoblasts and enhances the secretion of osteoproteoglycan by activating Ca-sensing receptors [36, 37]. Previously, it has been reported that the infiltration of Sr into bone scaffold materials increases the levels of early markers of osteoblast differentiation and ALP activity, while maintaining bone composition and strength [19]. In addition, Sr regulates bone resorption by preventing the differentiation of preosteoclasts into osteoclasts and inducing osteoclast apoptosis via RANKL [38, 39].

Si, another trace element, is positively associated with the early phases of bone formation. Within calcium phosphate cement, Si significantly enhances osteoblast differentiation *in vitro* [17]. Si has also been found to delay osteoclast differentiation and inhibit resorption activities. Hence, adding Si to biomaterials enhances their biological activity and bone formation ability *in vivo* [40].

Zn also promotes osteoblast differentiation and induces bone formation by enhancing ALP activity and extracellular matrix mineralization; in a role similar to Sr, it regulates osteoclast activity [14, 41]. In addition, Zn improves the viability, differentiation, and expression of osteogenic genes in rat bone marrow-derived pericytes as well as new bone formation in ovariectomized rabbit models *in vivo* [18]. Zn can also form advantageous boundaries with various types of bacteria by generating reactive oxygen species [42]. Coating titanium with Zn^2+^ not only promotes BMSCs differentiation but also exerts excellent antimicrobial effects against *Escherichia coli* and *Staphylococcus aureus* [16]. However, the effects of trace elements have been reported to be not only beneficial in enhancing osteoinductive properties and promoting osteogenesis but also cytotoxic. As for Sr, its cytotoxicity has been previously reported in vitro using rat BMSCs [38]. However, in this study, no significant cytotoxicity was observed at any of the concentrations and combinations used.

Compared to the previously reported method of doping trace elements into the material structure [14, 33, 43], we believe that nano-coating the surface of existing implants with trace elements offers the advantage of not impacting the mechanical and structural properties of the implants. Indeed, using SEM observation, our method of apatite coating maintained the structural integrity of β-TCP at high temperatures.

In the biochemical evaluation of β-TCP disks removed after 4 weeks of implantation *in vivo*, ALP activity and OC content, which are markers of osteogenic potential, were significantly increased in the apatite-coated groups. These results indicated that osteogenesis was effectively promoted. In these assays, there was no statistically significant difference between the SrZnSiP and SrSiP groups.

Similarly, Micro-CT-based radiological evaluation and histological observation showed that new bone formation was enhanced in the apatite coating groups. New bone formation areas were observed throughout the pores on β-TCP. These results indicated that apatite coating was able to sufficiently spread inside β-TCP and that it enhanced new bone formation.

*In vitro and in vivo*, qRT-PCR results revealed that the apatite-coated groups showed significantly increased mRNA levels of genes encoding ALP, OC, and BMP-2, all of which are indicators of osteoblast differentiation and mineralization. In addition, qRT-PCR results showed that the mRNA levels of the gene encoding VEGF, as an indicator of angiogenesis, were significantly increased. VEGF is a vital angiogenic factor that is predominantly produced in tissues that acquire new capillary networks. It contributes to the upregulation of BMP-2 expression in endothelial cells and demonstrates improved cross-reactivity in signaling pathways between endothelial and osteoblastic lineage cells [25]. In a previous study, Si has been also reported to promote angiogenesis [44]. Therefore, the present study suggests that synergistic effects of the different advantages of trace elements (Sr, Si, Zn) may further enhance the promotion of osteogenesis.

Only the *in vivo* qRT-PCR findings revealed that all mRNA levels were significantly higher in the SrZnSiP group. Considering the above statistically significantly results and the marked antimicrobial benefits of Zn, we presume that SrZnSiP may be more resistant to infection and thus more beneficial in clinical practice.

This study has several limitations. First, we examined the effects of apatite coating using only 2 mm thick β-TCP disks. Therefore, it is necessary to determine if apatite coating can confer similar osteoinductive properties on thicker or larger scaffolds. Second, a biomechanical evaluation was not completed. In the future, we plan to evaluate the biomechanical properties of the coating and test them using bone defect models, in accordance with the relevant guidelines. Third, the amount of apatite on β-TCP disks has not been measured. It is, therefore, necessary to consistently apply the same apatite coating techniques and accurately evaluate the quality of the apatite coating. Fourth, we did not assess cytotoxicity *in vivo*, although no harmful effects were observed histologically; in the future, it will be necessary to investigate whether any related adverse events occur following implantation.

## Conclusions

In this study, we demonstrated that coating trace elements amounts of Sr, Si, and Zn with apatite to β-TCP conferred osteoinductive properties to β-TCP disks *in vitro* and *in vivo.* Hence, this technique may improve the clinical results following orthopedic surgery using bone graft substitutes such as β-TCP.

## Data Availability

The datasets used during the present study are available from the corresponding author on reasonable request.

## Abbreviations

ALP: Alkaline phosphate
BMP-2: Bone morphogenetic protein-2
BMSCs: Bone marrow mesenchymal cells
EDS: Energy dispersive X-ray spectrometer
EDTA: Ethylenediaminetetraacetic acid
GAPDH: Glyceraldehyde-3-phosphate
HA: Hydroxyapatite
OC: Osteocalcin
PET: Polyethylene terephthalate
PEEK: Polyether-ether-ketone
qPCR: Quantitative polymerase chain reaction
SD: Standard deviation
SEM: Scanning electron microscopy
SrSiP: Silicate-substituted Sr apatite
SrZnSiP: Silicate- and Zn-co-substituted Sr apatite
VEGF: Vascular endothelial growth factor

## References

[1] Roberts TT, Rosenbaum AJ (2012). Bone grafts, bone substitutes and orthobiologics: the bridgebetween basic science and clinical advancements in fracture healing. Organogenesis.

[2] Okanoue Y (2012). Comparison of in vivo bioactivity and compressive strength of a novel superporous hydroxyapatite with beta-tricalcium phosphates. Arch Orthop Trauma Surg.

[3] Takeuchi R, Bito H, Akamatsu Y (2010). In vitro stability of open wedge high tibial osteotomy with synthetic bone graft. Knee.

[4] Putnis S (2020). The outcome of biphasic calcium phosphate bone substitute in a medial opening wedge high tibial osteotomy. J Mater Sci Mater Med.

[5] Aulakh TS (2009). Long-term clinical outcomes following the use of synthetic hydroxyapatite and bone graft in impaction in revision hip arthroplasty. Biomaterials.

[6] Tanaka T (2008). Bone formation and resorption in patients after implantation of beta-tricalcium phosphate blocks with 60 % and 75 % porosity in opening-wedge high tibial osteotomy. J Biomed Mater Res B Appl Biomater.

[7] Kira T (2017). Bone regeneration with osteogenic matrix cell sheet and tricalcium phosphate: An experimental study in sheep. World J Orthop.

[8] Ueha T (2015). Utility of tricalcium phosphate and osteogenic matrix cell sheet constructs for bone defect reconstruction. World J Stem Cells.

[9] Akahane M (2010). Scaffold-free cell sheet injection results in bone formation. J Tissue Eng Regen Med.

[10] Ohgushi H (1996). Osteogenic differentiation of cultured marrow stromal stem cells on the surface of bioactive glass ceramics. J Biomed Mat Res.

[11] Kawate K (2006). Tissue-engineered approach for the treatment of steroid-induced osteonecrosis of the femoral head: transplantation of autologous mesenchymal stem cells cultured with beta-tricalcium phosphate ceramics and free vascularized fibula. Artif Organs.

[12] Lindhorst D (2010). Effects of VEGF loading on scaffold-confined vascularization. J Biomed Mater Res A.

[13] Kim JW (2012). Volumetric bone regenerative efficacy of biphasic calcium phosphate-collagen composite block loaded with rhBMP-2 in vertical bone augmentation model of a rabbit calvarium. J Biomed Mater Res A.

[14] Fielding GA (2019). Regulation of osteogenic markers at late stage of osteoblast differentiation in silicon and zinc doped porous TCP. J Funct Biomater.

[15] James AW (2016). A review of the clinical side effects of bone morphogenetic protein-2. Tissue Eng Part B Rev.

[16] Hu H (2012). Antibacterial activity and increased bone marrow stem cell functions of Zn-incorporated TiO2 coatings on titanium. Acta Biomater.

[17] Mestres G, Le Van C, Ginebra MP (2012). Silicon-stabilized alpha-tricalcium phosphate and its use in a calcium phosphate cement: characterization and cell response. Acta Biomater.

[18] Yu J (2017). Zinc-modified calcium silicate coatings promote osteogenic differentiation through TGF-beta/Smad pathway and osseointegration in osteopenic rabbits. Sci Rep.

[19] Wang S (2019). Effect of strontium-containing on the properties of Mg-doped wollastonite bioceramic scaffolds. Biomed Eng Online.

[20] Furukawa A. The formation of strontium apatites through alkaline hydrolysis of strontium hydrogen phosphate and their crystallographic characterization. Ceramics Int. 2021. in press.

[21] Kawasaki S (2020). In vitro osteogenesis of rat bone marrow mesenchymal cells on PEEK disks with heat-fixed apatite by CO2 laser bonding. BMC Musculoskelet Disord.

[22] Egawa T (2019). Silicate-substituted strontium apatite nano coating improves osteogenesis around artificial ligament. BMC Musculoskelet Disord.

[23] Furukawa A, Akahane M, Tanaka Y (2018). CO2 laser bonding of silicate-substituted strontium apatite on PEEK and osteointegration on its surface. Key Eng Mat.

[24] Nakamura A (2010). Cell sheet transplantation of cultured mesenchymal stem cells enhances bone formation in a rat nonunion model. Bone.

[25] Nakano K (2016). Promotion of osteogenesis and angiogenesis in vascularized tissue-engineered bone using osteogenic matrix cell sheets. Plast Reconstr Surg.

[26] Akahane M (2008). Osteogenic matrix sheet-cell transplantation using osteoblastic cell sheet resulted in bone formation without scaffold at an ectopic site. J Tissue Eng Regen Med.

[27] Tohma Y (2008). Bone marrow-derived mesenchymal cells can rescue osteogenic capacity of devitalized autologous bone. J Tissue Eng Regen Med.

[28] Inagaki Y (2013). Osteogenic matrix cell sheet transplantation enhances early tendon graft to bone tunnel healing in rabbits. Biomed Res Int.

[29] Casarrubios L (2020). Silicon substituted hydroxyapatite/VEGF scaffolds stimulate bone regeneration in osteoporotic sheep. Acta Biomater.

[30] DeBaun MR (2019). Preclinical induced membrane model to evaluate synthetic implants for healing critical bone defects without autograft. J Orthop Res.

[31] Yeo A, Wong WJ, Teoh SH (2010). Surface modification of PCL-TCP scaffolds in rabbit calvaria defects: Evaluation of scaffold degradation profile, biomechanical properties and bone healing patterns. J Biomed Mater Res A.

[32] Lee SS (2013). Bone regeneration with low dose BMP-2 amplified by biomimetic supramolecular nanofibers within collagen scaffolds. Biomaterials.

[33] Ke D (2019). Effects of MgO, ZnO, SrO, and SiO2 in tricalcium phosphate scaffolds on in vitro gene expression and in vivo osteogenesis. Mater Sci Eng C Mater Biol Appl.

[34] Fielding GA, Bandyopadhyay A, Bose S (2012). Effects of silica and zinc oxide doping on mechanical and biological properties of 3D printed tricalcium phosphate tissue engineering scaffolds. Dent Mater.

[35] Li X (2009). The optimum zinc content in set calcium phosphate cement for promoting bone formation in vivo. Mater Sci Eng C Mater Biol Appl.

[36] Brennan TC (2009). Osteoblasts play key roles in the mechanisms of action of strontium ranelate. Br J Pharmacol.

[37] Lems WF, den Heijer M. Established and forthcoming drugs for the treatment of osteoporosis. Neth J Med. 2013;71(4):188-93. 23723112

[38] Khlusov IA (2014). Modulating effect of matrices with calcium phosphate coating on cytotoxicity of strontium ranelate and ibandronic acid in vitro. Bull Exp Biol Med.

[39] Tat SK (2011). Strontium ranelate inhibits key factors affecting bone remodeling in human osteoarthritic subchondral bone osteoblasts. Bone.

[40] Izquierdo-Barba I (2019). Synergistic effect of Si-hydroxyapatite coating and VEGF adsorption on Ti6Al4V-ELI scaffolds for bone regeneration in an osteoporotic bone environment. Acta Biomater.

[41] Tao B (2019). Zn-incorporation with graphene oxide on Ti substrates surface to improve osteogenic activity and inhibit bacterial adhesion. J Biomed Mater Res A.

[42] Sirelkhatim A (2015). Review on zinc oxide nanoparticles: antibacterial activity and toxicity mechanism. Nanomicro Lett.

[43] Bose S (2013). Understanding of dopant-induced osteogenesis and angiogenesis in calcium phosphate ceramics. Trends Biotechnol.

[44] Li H, Chang J (2013). Bioactive silicate materials stimulate angiogenesis in fibroblast and endothelial cell co-culture system through paracrine effect. Acta Biomater.

